# Lessons learned in 20 years of endoscopic endonasal surgery for pterygopalatine and infratemporal fossae lesions: analysis of a patient series and systematic review of literature

**DOI:** 10.3389/fonc.2025.1568913

**Published:** 2025-06-17

**Authors:** Matteo Zoli, Giacomo Sollini, Alessia Giorli, Alessandro Carretta, Marcello Magnani, Arianna Rustici, Martina Conti, Laura Maria Beatrice Belotti, Corrado Zenesini, Sofia Asioli, Paolo Farneti, Ernesto Pasquini, Diego Mazzatenta

**Affiliations:** ^1^ Programma Neurochirurgia Ipofisi - Pituitary Unit, IRCCS Istituto delle Scienze Neurologiche di Bologna, Bologna, Italy; ^2^ Department of Biomedical and Neuromotor Sciences (DIBINEM), University of Bologna, Bologna, Italy; ^3^ ENT Unit, Bellaria Hospital, Bologna, Italy; ^4^ Neuroradiology Unit, Ospedale Maggiore, IRCCS Istituto delle Scienze Neurologiche di Bologna, Bologna, Italy; ^5^ Epidemiology and Statistics Unit, IRCCS Istituto delle Scienze Neurologiche di Bologna, Bologna, Italy; ^6^ IRCCS Istituto delle Scienze Neurologiche di Bologna, Bologna, Italy

**Keywords:** pterygopalatine fossa, infratemporal fossa, endoscopic endonasal approach, skull base, malignancies, meningioma, juvenile angiofibroma, surgery

## Abstract

**Introduction:**

Recently, the endoscopic endonasal approach (EEA) has been proposed as a possible surgical option for benign and malignant tumors, located in the infratemporal (ITF) and pterygopalatine fossae (PPF). The aim of this study is to analyze the surgical outcome of the EEA for these lesions, identifying the preoperative factors affecting tumor resection.

**Materials and methods:**

All consecutive cases of PPF and ITF tumors operated through an EEA have been retrospectively collected. Preoperative clinical and radiological features, surgical outcome, complications and patient follow-up have been analyzed. A systematic review of literature has been performed.

**Results:**

The series includes 100 patients (66 males, 66.0%, mean age: 43.7 ± 22.1). The most common histotypes were juvenile angiofibromas (36 cases, 36.0%), malignancies (26, 26.0%), and chordomas (14, 14.0%). Gross total resection of the PPF/ITF portion of the tumor was achieved in 88 (88.0%) patients. The most common complication was represented by 10 cases (10.0%) of V2 hypoesthesia (3 transient). At logistic regression, tumor location in the temporo-masseteric and tubo-pharyngeal zones proved negatively associated with the GTR rate (p:0.05, p<0.01).

**Conclusion:**

EEA is an effective and safe approach for both benign and malignant tumors involving the PPF and ITF. It is characterized by a favorable complications rate and a quick patients recovery. We observed that the tumor extensions in the temporo-masseteric area and in the tubo-pharyngeal space were the most relevant factors negatively associated with complete tumor removal.

## Introduction

Neoplasms of pterygopalatine and infratemporal fossae (PPF and ITF) are a heterogenous group of tumors, which poses particular challenges to the surgeons for their deep location and intimate relationship with highly functional neurovascular and osteo-muscular structures (1–5).

Conventionally, they are surgically removed through open approaches, usually highly demolitive, with potentially significant neurological and functional sequelae and consequent detriment to patients’ quality of life (QoL) (2, 6, 7). A minimally invasive option is represented by the endoscopic endonasal approach (EEA), which allows us to access the PPF and ITF with the ventral corridor constituted by natural cavities, such as the nasal and paranasal sinuses (8–15). Indeed, these deep regions can be directly approached through the maxillary sinus, with a favorable angle of attack and a short distance from the nasal openings. The main advantages of this route is represented by its excellent and panoramic view, not requiring anyexternal bone drilling and neurovascular structures manipulation (15–18). Inded Multiple Authors have reported that this endoscopic transmaxillary-transpterygoid route could be effective for the surgical treatment of PPF and ITF lesions, such as encephaloceles, schwannomas, juvenile angiofibroma (JNA), meningiomas, but also loco-regional malignancies, preserving the osteo-ligamental and muscular structures with no visible scars, optimal cosmetic outcome, and consequently limited risk of permanent neurological or functional deficits, such as mastication or swallowing impairment (19–26). However, few reports have also pointed out the limitations of this route, which are mainly constituted by the difficulties to manage tumors with a very lateral or a deep extension toward the upper parapharyngeal space, or also extended to other lateral skull base regions, and by the lack of proximal control of the arterial and venous structures of the fossa (22, 27, 28). Moreover, a debate about its role in the management of malignancies is still open (22, 27, 28).

Therefore, indications of EEA for PPF and ITF tumors are still controversial and its effective role in clinical practice is discussed. The aim of this study is to analyze the surgical outcome and complications of EEA in our center and to determine its advantages and limits. A systematic review was also performed to consider the adoption and the outcome of this approach for PPF and ITF tumors in literature.

## Materials and methods

### Cases series

We present our retrospective case series of all consecutive patients with lesions involving the PPF and ITF surgically treated with an EEA in our Institution from 1998 to December 2022. Eligible patients were identified querying our institutional local database and then reviewing the neuroimaging and the clinical data for each case. Inclusion criteria consisted in 1. tumor location in the PPF and/or ITF, 2. surgical treatment with an EEA, 3. availability of pre- and postoperative clinical and neuroradiological data with a follow-up of at least 1 year. Cases of nasal-paranasal/skull base tumors not involving the PPF or ITF, or without adequate neuroimaging, or not undergone a purely endoscopic approach or with a follow-up shorter than 12 months were excluded.

Our management protocols for ITF and PPF have already been described elsewhere, as well as our surgical technique (29, 30). Briefly, all patients preoperatively underwent a complete neurological examination, considering their medical history with attention to previous surgical or adjuvant therapies for ITF and PPF tumors. ENT examination included a preoperative nasal endoscopy, with biopsy of the mass, when feasible. In all cases, preoperative imaging consisted in an MRI with contrast enhancement and a CT scan with angiographic sequences (CTA). Furthermore, patients suspected of a vascular lesion underwent a preoperative angiography with embolization 48–72 hours before the surgical procedure.

Surgery was carried out with patients in semi-sitting position, under general anesthesia, using HD 2D endoscopes (SPIES, Karl Storz, Tuttlingen, Germany, 4mm in diameter, 18 cm in length, with 0° and 30° scopes) with a high-definition camera, a neuronavigation system (StealthStation S7 and S8 MEDTRONIC, Louisville, CO. USA) with StealthMerge Software (MEDTRONIC, Louisville, CO. USA), intraoperative EcoDoppler (Mizuho 20 MHz Surgical Doppler System, Mizuho, Tokyo, Japan) and a 4 mm diamond high-speed drill (Midas Rex MR8, MEDTRONIC, Louisville, CO. USA). A 4-hands approach was performed, using a bi- or mono-nostril corridor, according to tumor extension.

**Figure 1:**
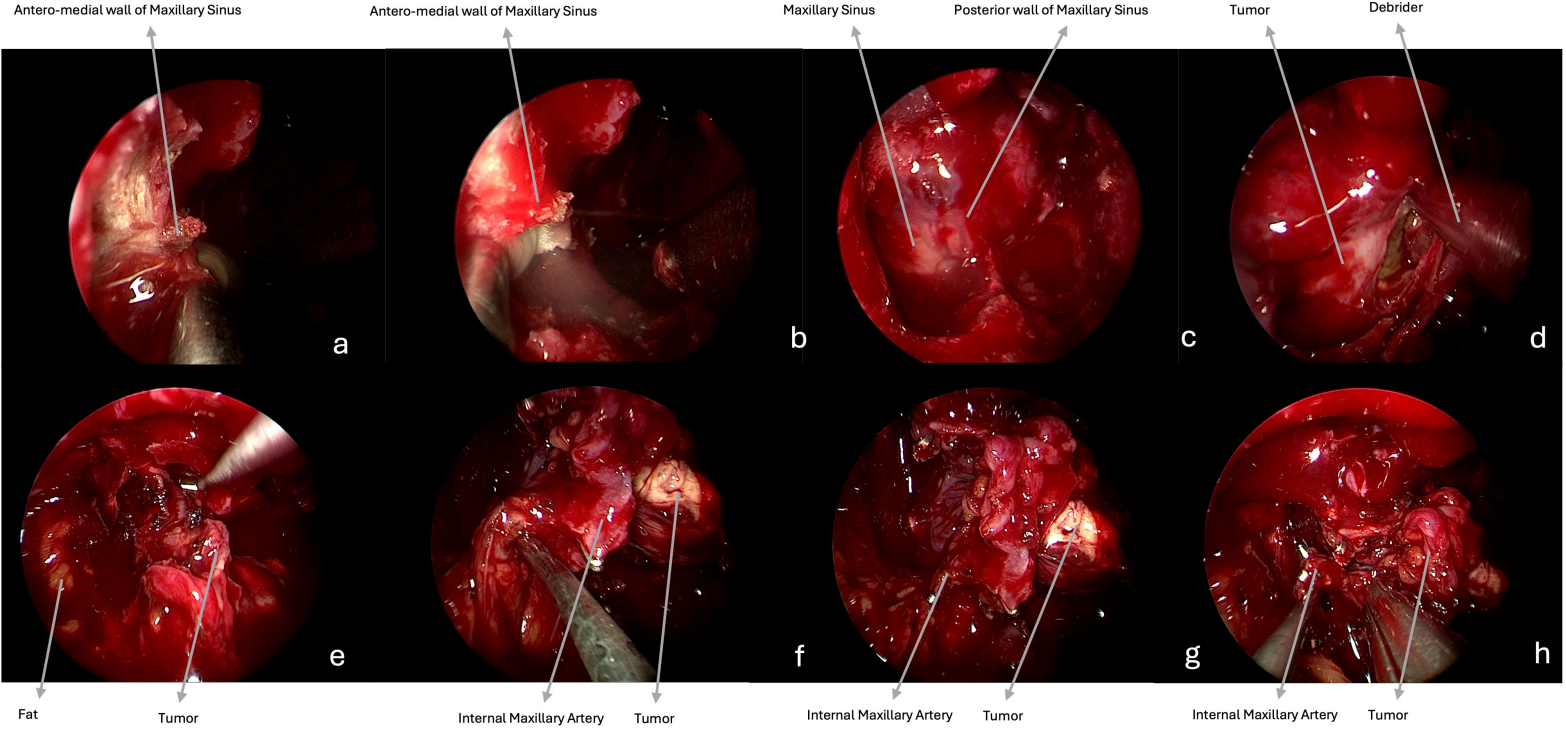
Intraoperative endoscopic images (0° scope). **(a–c)** The medial maxillectomy with a pre-lacrimal approach is performed to fully expose the posterior wall of the maxillary sinus. **(d)** After resection of this bony wall, the ventral access toward the PPF and ITF is obtained, and the tumor (meningioma) is visible. **(e)** The mass is progressively dissected from the surrounding structures and removed. **(f–h)** The internal maxillary artery is identified as proximally as possible, afterwards it is clipped and cut.

The first surgical steps are represented by an inferior complete or partial turbinectomy, followed by medial maxillectomy, whose anterior extension depends on the lateral tumor expansion (**Figure 1**). Afterward the perpendicular plate of the palatine bone is resected, the spheno-palatine artery is isolated and coagulated on exiting from the sphenopalatine foramen. A spheno-ethmoidectomy with drilling of the medial pterygoid plate is performed to increase the instruments’ maneuverability. After identification of the infraorbital nerve, dividing the projection of the PPF and ITF on the posterior wall of the maxillary sinus, the posterior wall of the maxillary sinus can be removed with high-speed drill or Kerrison rongeur to access the PPT and ITF (**Figure 1**).

**Figure 2:**
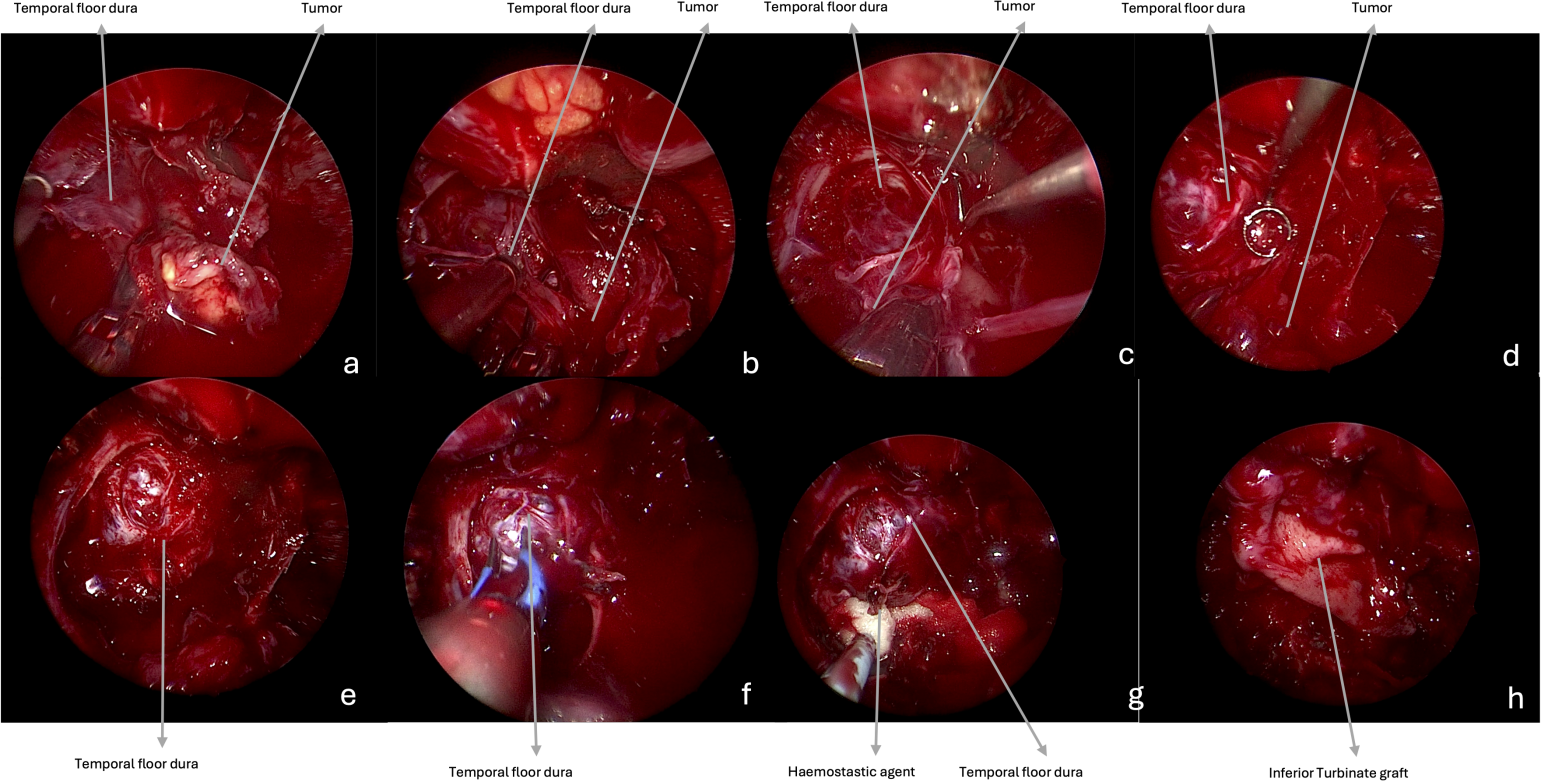
Intraoperative endoscopic images (0° scope). **(a–e)** The tumor is progressively resected, and the temporal floor dura is visible at the end of the removal. **(f, g)** Hemostasis is achieved with bipolar coagulation and with hemostatic agents. **(h)** Closure is performed with fat, covered by inferior turbinate graft.

Benign tumors are resected with a microsurgical technique, starting with a central debulking followed by cleavage from surrounding structures and with a centripetal removal of the tumor in large blocks up to negative surgical margins confirmed by frozen sections, for malignancies (**Figures 1**, **2**). Unless they are infiltrated by a malignancy, particular attention is paid to preserve the V2 and V3, which can be identified following their course respectively from the foramen rotundum and ovale. Moreover, V3 represents a helpful landmark for the petrosal tract of the internal carotid artery (ICA), which is the posterior limit of this approach. Internal maxillary artery and its branches are identified as soon as possible during tumor resection and then coagulated or clipped to avoid intra- or peri-operative bleeding. In case of CSF leak or exposure of the ICA, closure of the posterior wall of the maxillary sinus is performed with abdominal fat, covered by a mucoperiosteal graft, or an inferior turbinate flap previously harvested or bu other nasal flaps. Nasal cavities are packed with Merocel (MEDTRONIC, Louisville, CO. USA) (**Figure 2**).

Spontaneous breathing was restored at awakening with removal of oro-tracheal intubation and oral feeding within 6–8 hours after surgery. If no intraoperative CSF leak occurred, the patients could start a cautious mobilization the same day of surgery, otherwise 3 days of bed rest were prescribed. Patients were discharged after 3–4 days if no complications occurred.

Follow-up consisted in an endoscopic endonasal evaluation performed within 30 days from surgery. An MRI with contrast enhancement and a neurological evaluation was repeated before hospital discharge and at 3 months to assess the extension of tumor resection and patients’ clinical outcome. Afterwards, these examinations were repeated every 3–12 months, depending on the histopathological diagnosis. For malignancies, cases were discussed in a multidisciplinary team and, where indicated, adjuvant radiation or chemotherapies were advised.

**Figure 3:**
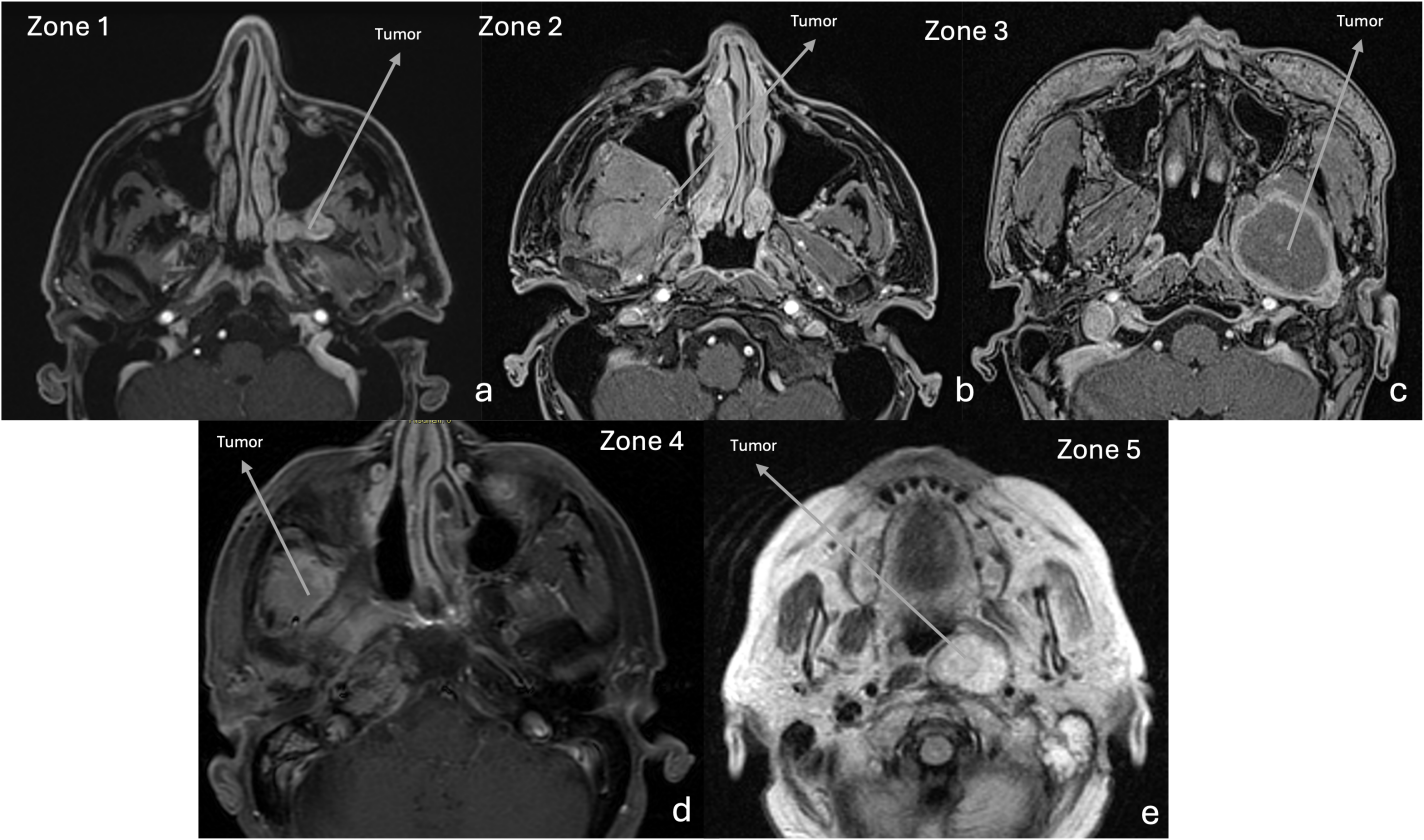
MRI. Axial T1-w after gadolinium, showing the tumor location, according to Li et al. ^26^ **(a)** Retromaxillary zone (zone 1). **(b)** Superior interpterygoid zone (zone 2). **(c)** Inferior interpterygoid zone (zone 3). **(d)** Temporo-masseteric zone (zone 4). **(e)** Tubopharyngeal zone (zone 5).

Patients’ preoperative clinical symptoms were collected based on medical records. On preoperative MRI, tumors were classified depending on their location as proposed by Li et al. (22). They were considered in the retromaxillary zone (zone 1), in case of location between the posterolateral wall of maxillary sinus and medial and lateral pterygoid and temporalis muscles complex; in the superior interpterygoid zone (zone 2), in case of location in the upper part of the ITF and PPF; in the inferior interpterygoid (zone 3), in case of location between the inferior head of the lateral pterygoid muscle, the medial pterygoid muscle and temporalis muscle; in the temporo-masseteric zone (zone 4), in case of location between the temporalis muscle and zygomatic arch; in the tubopharyngeal space (zone 5) in case of location in the upper part of the parapharyngeal space (**Figure 3**) (22).

At 3 months after postoperative MRI, radical resection was considered as gross total removal (GTR), in case of absence of visible tumor remnants, or as partial tumor removal (PTR) if a remnant is observed, and particular attention was paid to assess the location of this remnant inside or outside the PPF or IFT. Complications were considered, as well as their management, based on surgical reports. Recurrences or remnant progressions at follow-up, their treatment and timing were reported, as well as mortality due to the tumor progression.

### Statistical analysis

Our dataset of PPF and ITF tumors operated with a resective aim was analyzed to assess the clinical and neuroradiological factors associated with the GTR of the portion of the tumor located in these regions. Continuous variables were expressed as mean ± SD, while categorical variables as absolute (n) and relative frequency (%). A p-value < 0.05 was considered statistically significant. Mann-Chi-square or Fisher’s exact test were used to discrete variables and Whitney test or t-Student test for continuous ones. Multivariable logistic regression models were used to evaluate the associations with the variables significant in univariate analysis and the outcome. The results were presented as odds ratio (OR) and 95% confidence interval (95% CI).

Statistical analysis was performed using Stata (StataCorp. 2017. Stata Statistical Software: Release 15. College Station, TX: StataCorp LLC).

### Systematic review of literature

#### Search strategy

**Figure 4:**
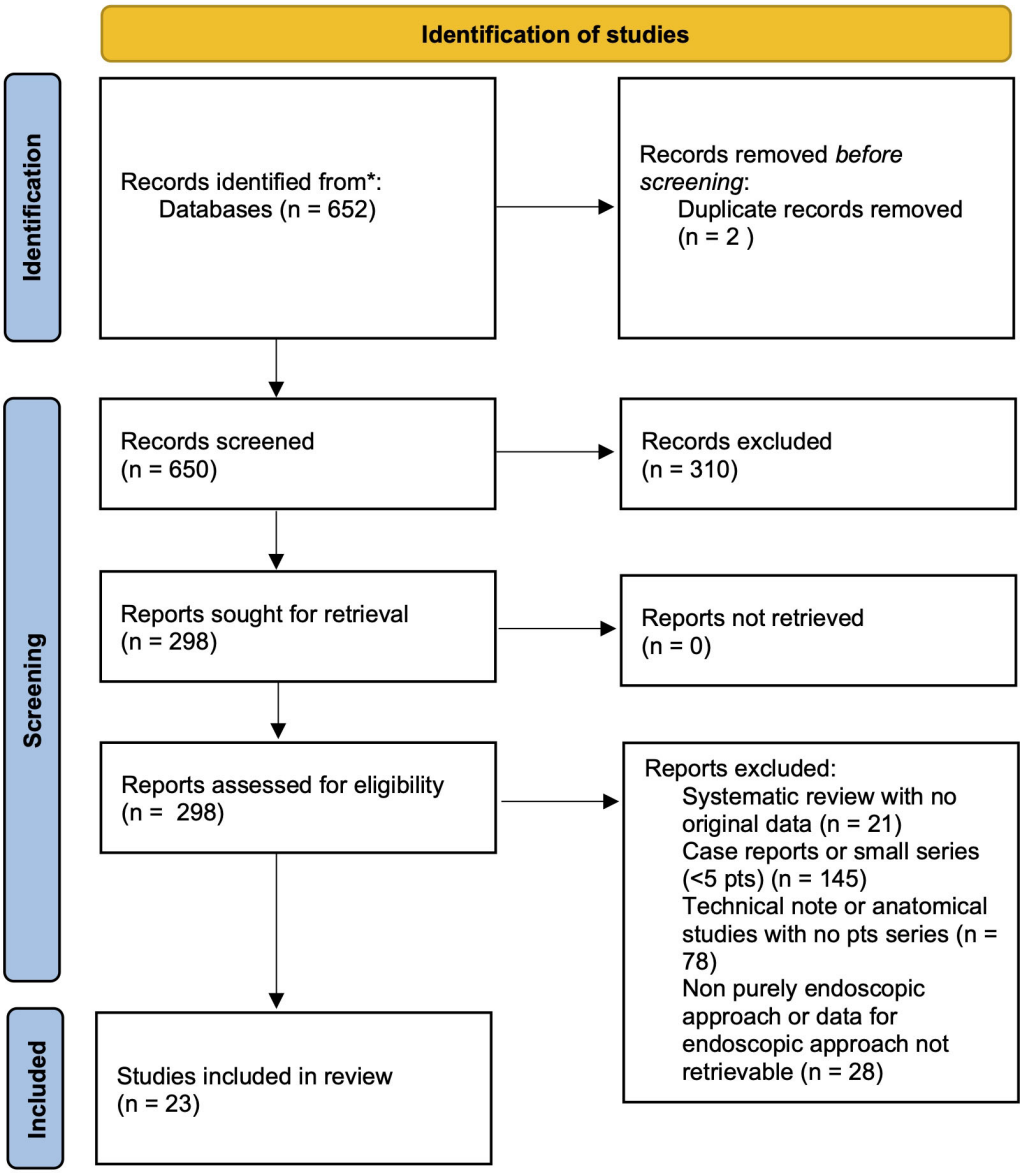
PRISM diagram of the systematic review of literature.

A systematic literature review was performed in accordance with the Preferred Reporting Items for Systematic Reviews and Meta‐analyses (PRISMA) statement guidelines (31). MEDLINE and Web of Science Core Collection (SCI-EXPANDED, SSCI, A&HCI, CPCI-S, CPCI-SSH, ESCI) databases were queried using individual keywords and MeSH terms. A purposely-defined search string was created for MEDLINE search: (“transmaxillary” [All Fields] OR “endoscopy” [MeSH Terms] OR “endoscopy” [All Fields] OR “endonasal” “[All Fields] OR “transnasal” “[All Fields] AND “pterygopalatine” [All Fields] OR “pterygomaxillary” “[All Fields] OR “infratemporal” “[All Fields]; WOS: TOPIC: (endoscopy or endoscopic) AND TOPIC: (infratemporal or pterygopalatine or pterygomaxillary). The results were then limited to the English language, human subjects and exclusively endoscopic approaches. After duplicate removal, title and abstracts were firstly screened and, for the papers deemed appropriate, full texts were obtained and reviewed for appropriateness and extraction of data. Article references were examined to identify any other relevant study (**Figure 4**).

#### Selection criteria

Only studies assessing the surgical outcomes of EEA for biopsy/resection of tumoral (benign or malignant) or pseudotumoral lesions of PPF or ITF were included (**Figure 4**). Exclusion criteria were: 1. combined or non-exclusive endoscopic endonasal approaches or endoscopy‐assisted procedures; 2. non-tumoral/pseudotumoral lesions; 3. less than 5 reported cases or if it was not possible to determine the extent of tumor removal after EEA for PPF or ITF tumors. Studies involving various surgical procedures or patient populations were included only if sufficient individual data on endoscopic removal of IFT and PPF tumors could be obtained to meet the inclusion criteria. Nonhuman, cadaveric and anatomical, technical, radiological and review studies, as well as papers with insufficient data were excluded. In case of aggregate (not-individual), missing and unreported data, the authors of each eligible study were contacted to obtain additional information.

#### Data extraction

Data from the included studies were extracted, organized and analyzed using Microsoft Excel 2019 (Microsoft Corp, Redmond, WA, USA).

## Results

### Cases series

Our series is composed of 100 patients (66 males, 66.0%, mean age was of 43.7 ± 22.1 years). Thirty-five patients (35.0%) were naïve, while 14 (14.0%) had already undergone surgery, 15 (15.0%) surgery followed by radio- and/or chemotherapy, 8 (10.9%) a neo-adjuvant treatment, 1 (1.0%) surgery and embolization, and other 27 (27.0%) JNA had been embolized before surgery (**Table 1**).

**Table 1:** Clinical presentation, surgical outcome, complications and follow-up.

Tumor	Age in yr. (Mean ± Std Dev.)	Sex	Prev. Treat.	Neur. Sympt.	Other non-neur. Sympt.	T. Location	Involved PPF/IFT zones	T. extension	PPF and/or ITF T. Res.	Compl.	Adjuvant Th.	Recur.	Recur. time (in mo.) (Mean ± Std Dev.)	Treatment of recur.	Deceased	Time of Dec. in mo. (Mean ± Std Dev.)
Meningioma and SFT (13 pts)	55.0 ± 8.3	12 Fem.; 1 Mal.	7 Surg. 3 Surg. + EBRT	4 Tr. Hypoesth.; 3 Tr. Neur.; 3 Dipl.; 3 Vis. Def.	7 Exopth.; 1 rhin. hypoac.; 1 malar swel.; 1 TMJ pain; 1 mast. dist.	1 PPF; 12 PPF + IFT	11 Zone 1; 12 Zone 2; 11 Zone 3; 9 Zone 4; 5 Zone 5	7 orbit; 13 MCF; 11 CS; 3 nas. f.; 4 PCF; 1 Petr. apex	6 GTR 7 PTR	4 V2 hypoth. (3 permanent, 1 transient)	8 EBRT (proton); 2 EBRT (photon); 2 radiosurg.	6	60.7 ± 63.0	2 eTOA; 2 craniot.; 1 EEA; 1 craniot.+ EBRT (proton)	3	89.5 ± 47.0
Chordoma (14 pts)	52.9 ± 12.8	7 Fem.: 7 Mal.	2 Surg.; 5 surg.+ EBRT; 1 radiosurg.	1 Tr. Neur.; 9 Dipl.; 1; 3 Vis. Def.; 1 Dysph.; 1 fac. palsy; 1 CN XII palsy	1 Nas. Obstr.	12 PPF; 2 PPF + IFT	11 Zone 1; 10 Zone 2; 1 Zone 3; 1 Zone 4; 1 Zone 5	6 MCF; 8 CS; 8 nas. f.; 3 PCF; 6 Petr. apex; 14 clivus; 1 rhinoph.	13 GTR 1 PTR	2 V2 hypoth. (1 permanent, 1 transient)	12 EBRT (proton); 1 CTx	13	35.4 ± 23.7	6 EEA; 1 EEA+EBRT (proton): 6 pall. care	13	75.7 ± 61
Chondrosarcoma (4 pts)	52.4 ± 10.6	2 Fem.; 2 Mal.	1 Surg.+ EBRT	1 Tr. Hypoesth.; 1 Tr. Neur.; 1 Dipl.	1 Nas. Obstr.	3 PPF; 1 PPF + IFT	3 Zone 1; 0 Zone 2; 0 Zone 3; 0 Zone 4; 1 Zone 5	4 CS; 2 Petr. apex; 2 clivus; 1 MC	3 GTR 1 PTR	None	2 EBRT (proton);	0	–	–	0	–
Schwannoma (4 pts)	53.1 ± 19.3	4 Fem.	1 Radiosurg.	1 Tr. Neur.; 1 Dipl.	1 Rhin. hypoac.;	2 PPF; 2 PPF + IFT;	2 Zone 1; 4 Zone 2; 3 Zone 3; 2 Zone 4; 0 Zone 5	1 CS; 1 nas. f.; 3 MC	4 GTR	3 permanent V2 hypoth.	None	0	–	–	0	–
JNA and hemangioma (36 pts)	21.6 ± 11.9	36 Mal.	1 Surg.+Emb; 27 Emb.	None	31 Nas. Obstr.; 28 Epist.; 1 Malar swel.	21 PPF; 3 IFT; 12 PPF + IFT	13 Zone 1; 16 Zone 2; 12 Zone 3; 9 Zone 4; 9 Zone 5	1 orbit; 1 MCF; 33 nas. f.; 22 rhinoph.; 8 sphenoidal sinus	35 GTR 1 PTR	1 transient V2 hypoesth.; 1 epist., 1 hard palate hemi-hypoesth.	None	1	26	1 EEA	0	–
Adenoidocystic C. (8 pts)	69.0 ± 9.0	1 Fem.; 7 Mal.;	2 Surg.; 2 Surg. + EBRT; 1 Surg. + radiosrug.; 1 EBRT; .	3 Tr. Neur.; 1 Vis. Def.	1 Exopth.; 2 Nas. Obstr.; 2 Epist.; 1 Rhin. Hypoac.;	1 PPF; 1 IFT; 6 PPF + IFT	6 Zone 1; 7 Zone 2; 4 Zone 3; 4 Zone 4; 4 Zone 5	3 orbit; 2 MCF; 3 CS; 6 nas. f.; 2 rhinoph.; 1 MC	8 GTR (6 RR 2 R1)	1 pansinusitis	3 EBRT (proton); 1 CTx.	3	25.8 ± 1.3	1 EEA; 1 EEA+EBRT (proton); 1 pall. care	3	21.4 ± 15.6
Inflammatory D. and inverted papilloma (3 pts)	49.3 ± 3.5	1 Fem.; 2 Mal.	None	1 Tr. Neur.;	1 Nas. Obstr.;	1 PPF; 1 IFT; 1 PPF + IFT	2 Zone 1; 1 Zone 2; 0 Zone 3; 0 Zone 4; 0 Zone 5	1 MCF; 1 CS; 1 nas. f.	1 GTR 2 Biopsy	None	2 corticoisteroid th.	0	–	–	0	–
Other malignancies (18 pts)	52.7 ± 22.7	7 Fem.; 11 Mal	3 Surg; 1 CTx; 1 EBRT; 3 CRT; 1 Surg + EBTR; 2 Surg. + CRT	3 Tr. Hypoesth; 6 Tr. Neur.; 4 Dipl.; 1 Vis. Def.	1 Exopth.; 6 Nas. Obstr.; 2 Epist.; 3 Rhin. hypoac.; 2 Retro-orbital/fac. pain	5 PPF; 2 IFT; 11 PPF + IFT	11 Zone 1; 14 Zone 2; 11 Zone 3; 4 Zone 4; 10 Zone 5	5 orbit; 4 MCF; 3 CS; 13 nas. f.; 1 clivus; 2 rhinoph.; 2 cervical space	18 GTR (12 RR, 6 R1)	3 Rhin. Hypoac.	2 EBRT (proton); 8 EBRT (photon); 5 CTx; 1 immunoth.	9	5.7 ± 10.1	1 open surgery + EBRT (proton); 8 pall. care	9	13.3 ± 12.4
Total (100 pts)	43.7 ± 22.1	34 Fem. (34.0%); 66 Mal. (66.0%);	35 (35.0%) Naïve; 14 (14.0%) Surg; 15 (15.0%) Surg + radio- and/or chemoth.; 1 Surg+Emb (1.0%) 8 (8.0%) radio- and/or chemoth.; 27 (27.0%) emb.	8 (8.0%) Tr. Hypoesth; 16 (16.0%) Tr. Neur.: 18 (18.0%) Dipl.; 8 (8.0%) Vis. def. 1 Dysph (1.0%); 1 (1.0%) Fac palsy; 1 (1.0%) XII CN palsy	9 (9.0%) exopth; 42 (42.0%) nas. obstr.; 32 (32.0%) epist.; 6 (6.0%) rhin. hypoac.; 2 (2.0%) malar swel.; 1 (1.0%), TMJ pain; 1 (1.0%) mast. dist. 2 (2.0%) retro-orbital/fac. pain;	46 (46.0%) PPF; 7 (7.0%) ITF; 47 (47.0%) PPF + ITF	59 (59.0%) Zone 1; 64 (64.0%) Zone 2; 42 (42.0%) Zone 3; 29 (29.0%) Zone 4; 30 (30.0%) Zone 5	16 (16.0%) orbit; 27 (27.0%) MCF; 31 (31.0%) CS; 65 (65.0%) nas. f.; 7 (7.0%) PCF; 9 (9.0%) petr. apex; 17 (17.0%) clivus; 27 (27.0%) rhinoph.; 5 (7.0%) MC; 8 sphenoid sinus (8.0%); 2 (2.0%) cervical space	88 (88.0%) GTR 10 (10.0%) PTR 2 (2.0%) Biopsy	10 (10%) V2 hypoesth. (7 permanent, 3 transient); 1 (1.0%) epist.; 1 (1.0%) hard palate hypoesth.; 1 (1.0%) pansinusitis; 3 (3.0%) rhin. hypoac.	27 (27.0%) EBRT (proton); 10 (10.0%) EBRT (photon); 2 (2.0%) radiosurg.; 7 (7.0%) CTx; 2 (2.0%) corticoisteroid th.; 1 (1.0%) immunoth	32 (32.0%)	32.2 ± 34.8	13 (13.0%) surg.; 4 (4.0%) surg.+ EBRT; 15 (15.0%) pall. care	28 (28.0%)	50.9 ± 54.1

Ad., adjuvant; CN, cranial nerve; Compl., complications; Craniot., craniotomy; CS, cavernous sinus; CTx, chemotherapy; Def., deficit; Dev., deviation; Dipl., diplopia; Dist., disturbances; Dysph., dysphagia; EBRT, external beam radiotherapy; EEA, endoscopic endonasal approach; Emb., Embolization; Epist., epistaxis; eTOA, endoscopic transorbital approach; Exopth., exophthalmos; Fac., facial; Fem., females; GTR, gross tumor removal; Hypoac., hypoacusia; Hypoesth., hyposthesia; ITF, infra temporal fossa; Mal., males; Mast., masticatory; MC, Meckel Cave; MCF, middle cranial fossa; Mo, months; Nas. f., nasal fossae; Nas. Obstr., nasal obstruction; Neur., neurological; Neur., neuralgia; Pall., palliative; PCF, posterior cranial fossa; Petr., petrous; PPF, pterygopalatine fossa; Prev., previous; PTR, partial tumor removal; Radiosur., radiosurgery; Recur., recurrence; Res., resection; Rhin, rhinogenic; Rhinoph., rhinopharynx; RR, radical resection with negative margins; R1, radical resection with positive margins; SFT, solitary fibrous tumor; Std, standard; Swel., swelling; Sympt., symptoms; T., tumor; Th., therapies TMJ, temporo-mandibular joint; Tr., trigeminal; Treat., treatment; Vis, visual; Yr, years.

The most common symptom was represented by nasal obstruction (42, 42.0%), followed by recurrent epistaxis (32, 32.0%), diplopia (18, 18.0%), trigeminal neuralgia (16, 16.0%) (**Table 1**). The majority of tumors were occupying both the PPF and ITF (47, 47.0%), while only 46 and 7 were exclusively located at the PPF or ITF, respectively. Neoplasms were located in the superior interpterygoid zone in 59 cases (59.0%), in the retromaxillary zone in 64 (64.0%), in the inferior interpterygoid in 42 (42.0%), in the temporo-masseteric zone in 29 (29.0%) and in tubo-pharyngeal zone in 30 cases (30.0%). The lesion was limited in the PPF or ITF only in 3 cases (3.0%), in all the others it was extended to the surrounding anatomical regions, as reported in **Table 1**. The most common histotypes were angiomas or juvenile nasal fibrangiomas (JNA) (36, 36.0%), malignant tumors (8 adenoidocystic carcinoma, 8.0%, and 18 other malignancies, 18.0%), followed by chordomas (14, 14.0%) and meningiomas/solitary fibrous tumors (13, 13.0%) (**Table 1**, **Supplementary Table 1**).

GTR of the PPF or ITF portion of the tumor was achieved in 88 patients (88.0%). Complications of the series consisted in 10 (10.0%) cases of *de novo* V2 hypoesthesia (7 permanent and 3 transient), 3 of postoperative rhinogenic hypoacusia (3.0%), 1 epistaxis (1.0%), 1 pansinusitis (1.0%), 1 permanent hard palate hemi-hypoesthesia (1.0%). An adjuvant treatment with radiation, chemo or immune-therapy was advised in 47 (47%) patients, in two pseudotumors a corticosteroid therapy was suggested after biopsy.

At follow-up (mean 39.9 months ± 46.0), pre-operative diplopia improved or normalized in 11 (61.1%), trigeminal neuralgia in 9 (56.3%), while trigeminal hypoesthesia was unchanged in 7 patients (87.5%) and improved only in one case (12.5%) (**Table 2**). Epistaxis resolved in all cases, nasal obstruction in 38 (90.5%) and proptosis in 8 (88.9%). Recurrences were observed in 32 cases (32.0%) after a mean of 32.2 ± 34.8, and at follow-up 28 (28.0%) were deceased for tumor progression.

**Table 2:** Patients clinical outcome at follow-up.

Type of Symptom	Symptom	Regres/impr.	Unchanged	Worsened
Neurological Symptoms	Trigeminal Hypoesthesia	1 (12.5%)	7 (87.5%)	0 (0%)
	Trigeminal Neuralgia	9 (56.3%)	7 (43.8%)	0 (0%)
	Diplopia	11 (61.1%)	7 (38.9%)	0 (0%)
	Visual deficit	5 (62.5%)	3 (37.5%	0 (0%)
	Dysphagia	1 (100%)	0 (0%)	0 (0%)
	Facial palsy	1 (100%)	0 (0%)	0 (0%)
	XII CN palsy	1 (100%)	0 (0%)	0 (0%)
Other symptoms	Exophthalmos	8 (88.9%)	1 (11.1%)	0 (0%)
	Nasal obstruction	38 (90.5%)	4 (9.5%)	0 (0%)
	Epistaxis	32 (100%)	0 (0%)	0 (0%)
	Rhinogenic hypoacusia	0 (0%)	6 (100%)	0 (0%)
	Malar swelling	1 (50%)	1 (50%)	0 (0%)
	TMJ pain	1 (100%)	0 (0%)	0 (0%)
	Masticatory Dist.	0 (0%)	1 (100%)	0 (0%)
	Retro-orbital or facial pain	2 (100%)	0 (0%)	0 (0%)

Dist., disturbance; Hypoesth., hypoesthesia; Impr., Improved; Regr., regressed; TMJ, temporo-mandibular joint.

### Statistical analysis

At univariate analysis, preoperative factors negatively associated with GTR of the tumor portion located in the PPF and ITF were its extension into the ITF (p:0.03) and the involvement of the inferior interpterygoid (zone 3), temporo-masseteric (zone 4) or tubo-pharyngeal (zone 5) regions (respectively p: 0.04, 0.01 and <0.01) (**Table 3**). At logistic regression, tumor extension into the temporo-masseteric (zone 4) and the tubo-pharyngeal zone (zone 5) confirmed their negative association with the GTR (p:0.05 and p<0.01) (**Table 3**).

**Table 3:** Statistical analysis.

Univariate Analysis	p
Sex	0.91
Age	0.43
Previous Treatment (surgical or not surgical)	0.72
Previous Surgery	0.35
Previous Radio/Chemo-therapy	0.26
Presence of neurological pre-operative symptoms	0.31
Presence of other non-neurological pre-operative symptoms	0.66
Tumor located in PPF	0.67
Tumor located in IFT	0.03
Involvement of retromaxillary zone (1)	0.56
Involvement of superior interpterygoid zone (2)	0.85
Involvement of inferior interpterygod zone (3)	0.04
Involvement of temporo-masseteric zone (4)	0.01
Involvement of tubo-pharingeal zone (5)	<0.01
Histotype	0.12
Logistic Regression	

	Odds Ratio	Sth. Err	z	p	95 Conf. Interv.
Tumor located in IFT	1.5	1.6	0.35	0.73	0.2 - 12.1
Involvement of inferior interpterygod zone (3)	0.3	0.4	-0.97	0.33	0.1 - 2.9
Involvement of temporo-masseteric zone (4)	6.4	6.3	1.92	0.05	0.9. 43.3
Involvement of tubo-pharingeal zone (5)	25.4	19.7	4.18	<0.01	5.6 - 116.2

### Systematic review of literature

We identified 23 studies presenting the results of EEA for 415 patients with PPF and/or ITF tumors (**Table 4**) (1, 4, 14, 32–51). Most of them were located in the PPF (345, 83,1%), while 205 (49.4%) were occupying the ITF. The most common histotype was represented by JNA or hemangiomas (300, 72.3%), followed by 34 (8.2%) schwannomas or other nerve sheet tumors and 13 (3.1%) meningiomas/fibrous dysplasia. Sixty-one (14.7%) cases of nasal/paranasal sinuses malignancies have been reported (16 ACC, 3.9%, and 45, 10.8%, other sinu-nasal malignancies).

**Table 4:** Systematic review of literature.

Authors	Year of pub.	N. cases	T. location	Histotype	Pre-operative sympt.	EOR	Compl.	Sympt. outcome	Local Recur/progr.	Compl. Th.	Mortality
Roger et al. (46)	2002	15	15 PPF, 3 ITF	15 JNA	NA	13 GTR, 2 STR	0	NA	0	None	0
Wormald et al. (48)	2003	6	6 PPF, 1 ITF	6 JNA	6 nas. obst.	6 GTR,	0	Nas. obst: 6 regr.	0	None	0
Hofmann et al. (41)	2005	19	19 PPF, 4 ITF	19 JNA	NA	14 GTR; 2 STR; 3 NA	0	NA	4	1 radiosurg.	0
Borghei et al. (35)	2006	9	9 PPF	9 JNA	NA	9 GTR	3 asympt. synechiae	NA	1	None	0
Gupta et al. (39)	2008	22	22 PPF, 2 ITF	22 JNA	NA	21 GTR, 1 STR	0	NA	0	None	0
Hofstetter et al. (42)	2010	7	6 PPF, 6 ITF	2 JNA, 1 chordoma, 1 ACC, 1 nas. glioma, 1 osteosarc., 1 lymph.	NA	3 GTR, 3 STR, 1 biopsy	3 sinusitis	NA	1 osteosarc.	1 RTx, 1 CRT, 2 CRx	2 (1 non related, 1 disease progression)
Nicolai et al. (44)	2010	41	38 PPF, 16 ITF	41 JNA	NA	38 GTR, 3 STR	0	NA	0	None	0
El Morsy et al. (36)	2011	15	15 PPF, 15 ITF	15 JNA	15 nas. obst., 15 epist.	15 GTR	1 transitory epiphora	NA	3	1 Radiosurg.	0
Al Sheibani et al. (32)	2011	20	21 PPF, 21 ITF	5 ACC, 9 SCC, 1 LE, 2 ADC, 2 MEC, 1 FDCC	NA	20 GTR (19 RR, 1 R1)	1 ICA injury	NA	7	19 RTx w or w/o CRx	18 (disease progression)
Zhang et al. (50)	2012	8	8 PPF, 8 ITF	8 schwannomas	1 nas. obst., 6 fac. numb., 2 fac. sens. disturb., 3 headache, 3 hypoac., 1 toothache, 1 dysosmia, 2 mastic. disturb., 3 hypopsia, 1 tinnitus	8 GTR	0	NA	0	None	0
Fyrmpas et al. (38)	2012	6	6 PPF, 1 ITF	6 JNA	6 nas. obstr.; 5 epist.	6 GTR	1 V2 hypostesia	Nas. Obstr.: 6 regr.; Epist.: 5 regr.	1	None	0
Taylor et al. (4)	2014	21	21 ITF	3 JNA, 1 chondrosarc., 3 ACC, 2 schwannomas, 1 meningioma, 2 IP, 1 PA, 1 GCT, 1 NMC, 1 pseudo t., 1 osteosarc., 1 Ewing sarc., 2 SCC, 1 lymph.	NA	16 GTR, 1 STR, 4 Bx	9 V2 numbness, 2 Eustachian tube dysfunction, 1 trismus	NA	3	3 RTx, 5 CRT, 1 CTx	2 (disease progression)
Battaglia et al. (1)	2014	37	20 PPF, 37 ITF	20 JNA, 1 hemangioma, 1 chondrosarc., 2 ACC, 2 schwannomas, 2 meningiomas, 1 SCC, 1 ADC, 1 MEC, 6 UCNT	NA	36 GTR, 1 STR	1 ICA injury, 4 facial numbness, 4 hearing loss, 1 CBHS, 1 dry eye, 3 temporary mast. dist.	NA	0	5 RTx, 3 CRT	0
Battaglia et al. (33)	2016	37	37 PPF	22 JNA, 1 hemangioma, 4 ACC, 2 schwannomas, 2 fibro-osseous dysplasia, 1 NEC, 1 ADC, 2 SCC, 1 NH 1 UCNT	31 nas. obstr.; 20 epist., 6 headache, 7 hypo-anosmia	37 GTR (36 RR, 1 R1)	4 check/palate numbness (1 transient), 1 dry eye	NA	0	3 RTx, 1 CRT	2 (disease progression)
Zhou et al. (51)	2016	7	7 PPF, 6 ITF	7 schwannomas	3 fac. numb., 3 headache, 1 diplopia	7 GTR	4 fac. numb. (3 transitory, 1 permanent)	NA	0	None	0
Janakiram et al. (43)	2016	15	15 PPF	15 JNA	15 nas. obstr., 15 epist.	13 GTR, 2 STR	3 synechiae	NA	1	NA	0
Plzak et al. (45)	2017	13	13 PPF, 8 ITF	10 JNA, 2 schwannoma, 1 SNUC	12 nas. obstr., 6 epist., 4 pain	12 GTR; 1 STR	8 V2 hypoesthesia (4 transient), 3 trismus (1 transient), 1 dry eye	NA	0	1 CRT (proton beam)	0
Shin et al. (47)	2018	6	6 PPF, 3 ITF	6 meningiomas	2 facial hypostesia, 2 exopht., 2 visual loss, 3 diplopia, 1 ptosis	5 GTR, 1 STR	1 transient V2 dysthesia, 1 dry eye	Visual loss: 1 unch., 1 regr., Hyposthesia: 1 unch.; 1 regr., Ptosis: 1 unch., Diplopia: 1 unch., 2 regr., Proptosis: 2 impr.	2	2 RTx, 1 radiosurg.	1 (disease progression)
Epprecht et al. (37)	2018	9	8 PPF, 2 ITF	9 JNA	2 hypoac.	5 GTR, 4 STR	3 rhynogenic hypoac.	Hypoac.: 2 unch.	1	None	0
Ozawa et al. (14)	2020	12	12 PPF, 5 ITF	2 JNA, 1 ACC, 1 schwannoma, 1 mal. ner. sheet t., 1 ganglioneuroma, 2 meningioma, 2 leiomyosarc., 1 carc., 1 lymph.	NA	3 GTR, 3 STR, 6 Bx	2 V2 hyposthesia (1 permanent, 1 transient)	NA	0	5 RTx (2 proton beam), 2 CTx	1 (disease progression)
Bignami et al. (34)	2022	57	33 PPF, 25 ITF	57 JNA	NA	56 GRT, 1 STR	NA	NA	0	None	0
Gupta et al. (40)	2022	25	25 PPF, 17 ITF	25 JNA	25 nas. obstr., 6 headache, 8 hypoac., 4 diplopia/exopht, 18 rhinorrhea, 12 rinolalia	23 GTR, 2 STR	0	NA	0	None	0
Wu et al. (49)	2023	8	4 PPF, 4 ITF	8 Schwannoma	7 fac. numb., 3 headache, 1 hypoac., 1 exopht, 1 mast dist., 1 visual dist., 1 tinnitus, 1 dizziness	8 GTR	1 CN VI palsy	Fac. numb.,: 6 impr., 1 unch.; Hypoacusia: 1 unch., Headache: 3 impr., Mast dist.: 1 impr., Tinnitus: 1 impr., Dizziness: 1 impr., Proptosis: 1 impr.; Visual dist.: 1 unch.	0	None	0
Total		415	345 (85.1%) PPF, 205 (49.4%) ITF	300 (72.3%) JNA or hemangiomas; 3 (0.7%) chordomas/chondrosarc; 16 (3.9%) ACC; 34 (8.2%) benign or malign nerve sheet tumors; 13 (3.1%) meningiomas/fibro-osseus dysplasia; 2 (0.5%) IP; 1 (0.2%) pseudo T.; 1 (0.2%) PA; 45 (10.8%) other sinunasal malignancies	111(26.7%) nas. obstr.; 67 (16.1%) epist.; 20 (4.8%) facial numbeness/hypoesth; 21 (5.1%) headache; 14 (3.4%) hypoac.; 5 (1.2%) facial pain; 8 (1.9%) dysosmia; 7 (1.7%) exopht., 3 (0.7%) mast dist.; 6 (1.4%) visual dist.; 2 (0.5%) tinnitus; 1 (0.2%) ptosis; 8 (1.9%) diplopia; 18 (4.3%) rhinorrhea; 12 (2.9%) rinolalia; 1 (0.2%) dizziness	374 (90.1%) GTR; 27(6.5%) STR; 11 (2.6%) Bsx	6 (1.4%) syniechiae; 3 (0.7%) sinusitis; 1 (0.2%) epiphora; 2 (0.5%) ICA injury; 33 (7.9%) V2 hypoesth. (10 transient); 9 (2.2%) eustachian tube dysf./hypoac. 4 (1.0%) trismus (1 transient); 1 (0.2%) CBHS; 4 (1.0%) dry eye; 3 (0.7%) transitory mast. dist;. 1 (0.2%) CN VI	Nas obs: 12 regr.; Epist.: 5 regr. Hyposthesia: 2 unch.; 7 regr./impr., Headache: 3 impr., Hypoac.: 1 unch., Proptosis: 3 impr., Mast. Dist.: 1 impr., Visual def.: 2 unch., 1 regr., Tinnitus: 1 impr., Ptosis: 1 unch., Diplopia: 1 unch., 2 regr., Dizziness: 1 impr.	24 (5.8%)	3 radiosusg.; 19 RTx, 19 RTx with or without Rx, 11 CRT. 5 CRx	26 (6.3%) (25 -6.0%- due to disease progression)

ACC, adenodocystic carcinoma; ADC, Adenocarcinoma; Carc., carcinoma; CBHS, Claude Bernard Horner Syndrome; Compl., complementary; Compl., complications; CRT, chemoradiotherapy; CTx, chemotherapy; Dist., disturbance; EOR, extent of resection; Epist., Epistaxis; Esthesioneurobl, estesioneuroblastoma; Fac. Numb., facial numbness; FDCC, follicular dendritic cell carcinoma; GCT, granular cell tumor; GTR, gross tumor removal; Hear. Loss, hearing loss; Hyperlacr., hyperlacrimation; Hypoac., hypoacusia; IFT, infratemporal fossa; Impr., improved; IP, inverted papilloma; JNA, juvenile naso-angiofibroma; LE, lymphoepitelioma; Lymph, lymphoma; Mal, malignant; Mast., masticatory; MEC., mucoepitheliod carcinoma; N., number; NA, not available; Nas. Obst., nasal obstruction; NEC, neuroendocrine carcinoma; Ner., nerve; NMC, nasopharyngeal mucoepidermoid carcinoma; Osteoradionecr., osteoradionecrosis; PA, pleormophic adenoma; PitNET, pituitary neuroendocrine tumor/adenoma; PPF, pterygopalatine fossa; Progr., progression; Pub., publication; Radiosurg., radiosurgery; Recur., recurrence; Regr., regressed; RR, radical resection with negative margins; R1, radical resection with positve margins; RTx, radiotherapy; Sarc., sarcoma; SCC, squamous cell carcinoma; Sens., sensory; SNUC, Undifferentiated sino-nasal carcinoma; STR, subtotal tumor removal; Sympt., symptoms; T., tumor; Th., therapy; UCNT, Undifferentiated Carcinoma of nasopharyngeal tract; Unch., unchanged.

The most common symptoms were represented by nasal obstruction (111, 26.7%) and recurrent epistaxis (67, 16.1%), neurological symptoms were mostly constituted by trigeminal hypoesthesia (20, 4.8%).

GTR was achieved in 374 (90.1%) patients. Complications consisted in synechiae (6, 1.4%), sinusitis (3, 0.7%), ICA blow-out (2, 0.5%), transient or permanent trigeminal hypoesthesia (33, 7.9%), eustachian tube dysfunction and rhinogenic hypoacusia (9, 2.2%), trismus (4, 1.0%), Claude Bernard Horner Syndrome (1, 0.2%), dry eye (4, 1.0%), temporary masticatory impairment (3, 0.7%), CN VI palsy (1, 0.2%), transitory epiphora due to naso-lacrimal duct injury (1, 0.2%).

Twenty-four (5.8%) local recurrences have been observed and 26 patients (6.3%) were deceased at follow-up (25, 6.0% for tumor progression).

## Discussion

In our large series of 100 cases, we have observed that EEA permits to effectively manage a large number of different PPF and ITF tumors with an excellent GTR (88.0%) and a favorable morbidity rate. Our results have been confirmed by the largest reported series: indeed, in the our review of literature, GTR was achieved in 90.1% of 415 cases, and the complication rate was estimated at 16.0%, with few permanent sequelae or potentially life-threatening events (1, 4, 14, 32–51).

It is interesting to observe that in the early studies in literature, the vast majority of PPF and ITF lesions treated with an EEA were represented by fibrovascular lesions as JNA, which are the 36% in our series (35, 39, 41, 44, 46, 48, 52). These tumors commonly extend into the PPF and/or ITF because of their multilobular shape, invading neural foramina or other skull base fissures from its point of origin in the spheno-palatine foramen or, according to other Authors, at the external opening of the pterygopalatine canal (35, 39, 41, 44, 46, 48, 52). The increasing experience in endoscopic treatment of sinonasal diseases, including loco-regional neoplasms, has brought many surgeons in the ‘00 to resect selected cases of JNA with such approach (35, 39, 41, 44, 46, 48, 52). The contemporary anatomical definition of the prelacrimal or Denker’s corridor to the maxillary sinus and consequently to the PPF and ITF has allowed worldwide surgeons to include more complex and invasive cases of JNA, achieving a tumor resection rate comparable to the open approaches, but with an inferior blood loss, limited cosmetic deformities, quick recovery, minimal morbidity and consequently reduced time of hospitalization (1, 4, 14, 32–53). Thereafter, thanks to the routinary adoption of the 4-hands technique, with the endoscope held by the second surgeon or a mechanical device, various benign and malignant PPF and ITF histotypes have been progressively considered suitable for EEA (1, 4, 5, 14, 32–34, 36–38, 40, 43, 45, 47, 49–51, 54–57). Indeed, the coupling of the microsurgical two-hands tumor removal technique with the dynamic and magnified surgical view of the endoscopic vision can favor the identification of the mass limits and boundaries, facilitating its complete resection (58). Consequently, as reported by Ozawa et al., along the years the number of nasal/paranasal sinuses malignancies treated with this approach has increased, representing, also, a large group of patients in our series (26%) (14). Indeed, EEA has proven to warrant a radical tumor resection with negative margins in a significant number of cases (75% of ACC and 66.7% of the other malignancies in our experience), allowing us to follow tumor infiltration inside the PPF and ITF. Moreover, thanks to the multidisciplinary collaboration between ENT and neurosurgeons, this route has been adopted also for other skull base tumors invading the PPF and ITF (57, 58). Particularly, it can be combined with other endoscopic endonasal corridors, i.e., the transclival corridor, to maximize the e resection of tumors such as chordomas and chondrosarcomas that originate respectively from the clivus or the petro-clival junction and frequently bulkily occupy the PPF and ITF (57). The direct approach given by the EEA allows the surgeon to expose the entire extension of these lesion, improving the chances to achieve a GTR (observed in 92.8% of chordomas and 75% of chondrosarcomas in our experience). Similarly, benign masses such as meningiomas or schwannomas, which can originate directly from the PPF or ITF or extend into these cavities from the surrounding skull base or cranial regions, such as middle or posterior fossa, cavernous sinus or Meckel cave, can be a potential target for the EEA (49, 51, 56, 59). Indeed, this approach gives a sufficient maneuverability to perform a initially bimanual central debulking of these benign lesions, later followed by their progressive cleavage from the surrounding structures, leading to a GTR of 46.1% for the meningiomas and of 100% for the schwannomas of our series. This different outcome can be explained considering the different consistencies of these lesions (usually firmer for meningiomas) and because of the different invasiveness of the surrounding soft and bony structures of these histotypes (49, 51, 56, 59).

One of the main limits of this approach has been represented by the poor control of arterial vessels in this area, mainly the internal maxillary artery and its branches, and the ICA (47). Therefore, for highly vascularized tumors (i.e., JNA, meningiomas, solitary fibrous tumors) the possibility of a preoperative embolization has to be considered to quicken the surgical procedures and reduce the intraoperative blood loss (47, 60). In our experience, we did not find significant difficulties in identifying tumor margins after embolization, which could represent a possible drawback of this procedure. Intraoperatively, we suggest identifying and clipping or ligating as early as possible the internal maxillary artery to reduce the risk of perioperative bleeding. Moreover, peculiar attention should be paid to the anatomical identification and preservation of the ICA, which can be encountered in the resection of large invasive skull base tumors (29, 30). In literature, two (0.5%) cases of ICA blow-out have been reported (1, 32). In our experience the main advice to prevent such complications is to identify its anatomical landmarks (i.e., V3, which lies anteriorly to the petrous tract of the ICA; the vidian nerve, which marks its petro-clival bend; the eustachian tube, which represents a reliable anatomical marker for its parapharyngeal tract with its bony insertion just anterior to the ICA canal) and by the routine adoption of technological devices such as neuronavigation and intraoperative Doppler to localize its course in real time (29, 30, 61).

A further limitation of EEA is represented by a lateral extension of the tumor into the surrounding facial spaces, cervical region, or the petrous bone, which would require an alternative external route or a combination of different complementary surgical approaches (61, 62). Moreover, we found that the classification proposed by Li et al, dividing the PPF and ITF into 5 regions (1:retromaxillary zone; 2: superior interpterygoid zone; 3: inferior interpterygoid; 4: temporo-masseteric zone; 5: tubopharyngeal space, schematically represented in **Figure 3**) can be helpful to predict the surgical outcome (22). Indeed, we observed that the lesion extension in the temporo-masseteric area or in the tubo-pharyngeal region (including in the upper pharyngeal space) represent the most significant negative prognostic factors for tumor resection (22). The temporo-masseteric zone is very lateral, bound externally by the mandible ramus (30). To reach such tumoral extension, it is of paramount importance to expand as anteriorly as possible the medial maxillectomy, to increase the surgical angle available for the ITF (5). Similarly, the involvement of the tubo-pharyngeal region hampers the surgical resection for the risk of damaging neurovascular and functional structures of the region (5). A possible alternative is represented by the adoption of an anterior transmaxillary approach, as a Caldwell-Luc procedure (ipsilateral or contralateral) to enlarge the exposure of the most lateral or deep tumor extension, however the specific morbidity of this approach should be considered (26). This confirms that the careful pre-operative evaluation of the tumor location and its extension into the PPF or ITF represents the most important factor to determine the complete tumor resection.

One of the main caveats of this approach is represented by the risk of V2 damage, with a permanent or transitory rate of postoperative facial hypoesthesia respectively of 5.5% and 2.4% in literature (30). The early identification of the nerve and its cleavage from the tumor mass, when not infiltrated, can significantly reduce this risk, and also contributing to the control of preoperative trigeminal neuralgia, which was observed in 56.3% of cases in our series (30). However, in case of neural infiltration by a malignant tumor, its sacrifice is mandatory (30).

In this study, we present our 20 years surgical experience, along this long time-span our technique has been progressively implemented, adapting the medial maxillectomy to each case extension, and progressively pushing the edge of envelope toward more challenging benign or malignant tumors. Moreover, we would like to remark that the endoscopic transmaxillary-pterygoid approach to PPF and ITF tumors requires a long training, and some key success factors are represented by surgeons specific experience and skills in endoscopic surgery, by an accurate case selection, and by the possibility of a multidisciplinary collaboration with dedicated ENT, neurosurgeons and other specialties involved in the management of these cases.

The limits of this study are represented by its retrospective design and by the low number of cases enrolled due to the rarity of the neoplasms of these regions. For these reasons, we have decided to include heterogenous histotypes, encompassing benign and malignant tumors. This has allowed us to give the most accurate and real-life description of the state of the art of EEA for PPF and ITF tumors, but, nevertheless, no definite conclusions on the long-term results could be drawn due to the small size of each group of neoplasms. Future studies with larger series could be helpful to determine the surgical outcome and the neurological results of each histotypes after this approach.

## Conclusions

After 20 years of experience, we report the effectiveness of the EEA to manage the majority of tumors involving the PPF and ITF. This minimally invasive approach is characterized by a satisfactory rate of tumor removal with a low morbidity, especially in terms of neurological deficits and functional sequelae, however it requires a long experience in endoscopic surgery.

In our study, the factors negatively associated with a complete tumor removal were the lateral extension of the tumor in the temporo-masseteric area or its involvement of the tubo-pharyngeal space. At the same time, the tumor’s more lateral or more inferior expansion into the facial or cervical spaces or the involvement of the petrous bone should be considered as the most significant limits of this approach.

Therefore, an accurate preoperative surgical planning, considering the tumor location and its extension, the multidisciplinary collaboration and the specific training in endoscopic skull base surgery, represent the most relevant key success factors for this approach.

## Data Availability

The raw data supporting the conclusions of this article will be made available by the authors, without undue reservation.

## References

[1] Battaglia P Turri-Zanoni M Dallan I Gallo S Sica E Padoan G. Endoscopic endonasal transpterygoid transmaxillary approach to the infratemporal and upper parapharyngeal tumors. Otolaryngol Head Neck Surg. (2014) 150:696–702. doi: 10.1177/0194599813520290 24457630

[2] Bradley PJ. Infratemporal fossa surgical approaches to primary/recurrent Malignancies of salivary origin: paradigm surgical shift, patient selection, and oncologic outcomes. Curr Opin Otolaryngol Head Neck Surg. (2020) 28:79–89. doi: 10.1097/MOO.0000000000000613 32011396

[3] Chung HJ Moon IS Cho H-J Kim C-H Sharhan SSA Chang JH. Analysis of surgical approaches to skull base tumors involving the pterygopalatine and infratemporal fossa. J Craniofac Surg. (2019) 30:589–95. doi: 10.1097/SCS.0000000000005108 30640855

[4] Taylor RJ Patel MR Wheless SA McKinney KA Stadler ME Sasaki-Adams D. Endoscopic endonasal approaches to infratemporal fossa tumors: a classification system and case series. Laryngoscope. (2014) 124:2443–50. doi: 10.1002/lary.24638 25513678

[5] Yafit D Duek I Abu-Ghanem S Ungar OJ Wengier A Moshe-Levyn H. Surgical approaches for infratemporal fossa tumor resection: Fifteen years’ experience of a single center. Head Neck. (2019) 41:3755–63. doi: 10.1002/hed.25906 31407445

[6] Sekhar LN Schramm VL Jones NF. Subtemporal-preauricular infratemporal fossa approach to large lateral and posterior cranial base neoplasms. J Neurosurg. (1987) 67:488–99. doi: 10.3171/jns.1987.67.4.0488 3655886

[7] Zanoletti E Mazzoni A Martini A Abbritti RV Albertini R Alexandre E. Surgery of the lateral skull base: a 50-year endeavour. Acta Otorhinolaryngol Ital. (2019) 39:S1–S146. doi: 10.14639/0392-100X-suppl.1-39-2019 31130732 PMC6540636

[8] Abuzayed B Tanriover N Gazioglu N Cetin G Akar Z. Extended endoscopic endonasal approach to the pterygopalatine fossa: anatomic study. J Neurosurg Sci. (2009) 53:37–44. 19546841

[9] Cavallo LM Messina A Gardner P Esposito F Kassam AB Cappabianca P. Extended endoscopic endonasal approach to the pterygopalatine fossa: anatomical study and clinical considerations. Neurosurg Focus. (2005) 19:E5. doi: 10.3171/foc.2005.19.1.6 16078819

[10] Karkas A Zimmer LA Theodosopoulos PV Keller JT Prades J-M. Endonasal endoscopic approach to the pterygopalatine and infratemporal fossae. Eur Ann Otorhinolaryngol Head Neck Dis. (2021) 138:391–5. doi: 10.1016/j.anorl.2020.12.009 33384280

[11] Kassam AB Gardner P Snyderman C Mintz A Carrau R. Expanded endonasal approach: fully endoscopic, completely transnasal approach to the middle third of the clivus, petrous bone, middle cranial fossa, and infratemporal fossa. Neurosurg Focus. (2005) 19:E6. doi: 10.3171/foc.2005.19.1.7 16078820

[12] de Lara D Ditzel Filho LFS Prevedello DM Carrau RL Kasemsiri P Otto BA. Endonasal endoscopic approaches to the paramedian skull base. World Neurosurg. (2014) 82:S121–129. doi: 10.1016/j.wneu.2014.07.036 25496623

[13] Oakley GM Harvey RJ. Endoscopic resection of pterygopalatine fossa and infratemporal fossa Malignancies. Otolaryngol Clin North Am. (2017) 50:301–13. doi: 10.1016/j.otc.2016.12.007 28162242

[14] Ozawa H Sekimizu M Saito S Nakamura S Mikoshiba T Toda M. Endoscopic endonasal management of pterygopalatine fossa tumors. J Craniofac Surg. (2021) 32:e454–7. doi: 10.1097/SCS.0000000000007292 33252536

[15] Rivera-Serrano CM Terre-Falcon R Fernandez-Miranda J Prevedello D Snyderman CH Gardner P. Endoscopic endonasal dissection of the pterygopalatine fossa, infratemporal fossa, and post-styloid compartment. Anatomical relationships and importance of eustachian tube in the endoscopic skull base surgery. Laryngoscope. (2010) 120 Suppl 4:S244. doi: 10.1002/lary.21711 21225842

[16] Alfieri A Jho H-D Schettino R Tschabitscher M. Endoscopic endonasal approach to the pterygopalatine fossa: anatomic study. Neurosurgery. (2003) 52:374–378; discussion 378-380. doi: 10.1227/01.neu.0000044562.73763.00 12535367

[17] Fahmy CE Carrau R Kirsch C Meeks D de Lara D Solares CA. Volumetric analysis of endoscopic and traditional surgical approaches to the infratemporal fossa. Laryngoscope. (2014) 124:1090–6. doi: 10.1002/lary.24428 24114920

[18] Hartnick CJ Myseros JS Myer CM. Endoscopic access to the infratemporal fossa and skull base: a cadaveric study. Arch Otolaryngol Head Neck Surg. (2001) 127:1325–7. doi: 10.1001/archotol.127.11.1325 11701068

[19] Finger G Gun R Wu KC Carrau RL Prevedello DM. Endoscopic endonasal transpterygoid approach: technical lessons. Oper Neurosurg (Hagerstown). (2023) 25:e272. doi: 10.1227/ons.0000000000000738 37350591

[20] Fortes FSG Sennes LU Carrau RL Brito R Ribas GC Yasuda A. Endoscopic anatomy of the pterygopalatine fossa and the transpterygoid approach: development of a surgical instruction model. Laryngoscope. (2008) 118:44–9. doi: 10.1097/MLG.0b013e318155a492 17989582

[21] Kim SM Paek SH Lee JH. Infratemporal fossa approach: the modified zygomatico-transmandibular approach. Maxillofac Plast Reconstr Surg. (2019) 41:3. doi: 10.1186/s40902-018-0185-x 30687683 PMC6331346

[22] Li L London NR Prevedello DM Carrau RL. Anatomy based corridors to the infratemporal fossa: Implications for endoscopic approaches. Head Neck. (2020) 42:846–53. doi: 10.1002/hed.26055 PMC9292508 31880379

[23] Pacino GA Redondo LM Cocuzza S Maniaci A Da Mosto MC Boscolo-Rizzo P. Primary hemangiopericytoma of the infratemporal fossa. J Biol Regul Homeost Agents. (2020) 34:691–5. doi: 10.23812/19-431-L 32431142

[24] Park HH Hong SD Kim YH Hong C-K Woo KI Yun I-S. Endoscopic transorbital and endonasal approach for trigeminal schwannomas: a retrospective multicenter analysis (KOSEN-005). J Neurosurg. (2020) 133:467–76. doi: 10.3171/2019.3.JNS19492 31226689

[25] Raza SM Amine MA Anand V Schwartz TH. Endoscopic endonasal resection of trigeminal schwannomas. Neurosurg Clin N Am. (2015) 26:473–9. doi: 10.1016/j.nec.2015.03.010 26141365

[26] Theodosopoulos PV Guthikonda B Brescia A Keller JT Zimmer LA. Endoscopic approach to the infratemporal fossa: anatomic study. Neurosurgery. (2010) 66:196–202; discussion 202-203. doi: 10.1227/01.NEU.0000359224.75185.43 20023550

[27] Kassam AB Vescan AD Carrau RL Prevedello DM Gardner P Mintz AH. Expanded endonasal approach: vidian canal as a landmark to the petrous internal carotid artery. J Neurosurg. (2008) 108:177–83. doi: 10.3171/JNS/2008/108/01/0177 18173330

[28] Nicolai P Battaglia P Bignami M Bolzoni Villaret A Delù G Khrais T. Endoscopic surgery for Malignant tumors of the sinonasal tract and adjacent skull base: a 10-year experience. Am J Rhinol. (2008) 22:308–16. doi: 10.2500/ajr.2008.22.3170 18588765

[29] Zoli M Sollini G Rustici A Carretta A Magnani M Guaraldi F. Combined endoscopic transorbital and transmaxillary-pterygoid approach for a recurrent spheno-orbital meningioma: 2-dimensional operative video. Oper Neurosurg (Hagerstown). (2024) 27:258–9. doi: 10.1227/ons.0000000000001110 38442482

[30] Zoli M Sollini G Zaccagna F Fabbri VP Cirignotta L Rustici A. Infra-temporal and pterygo-palatine fossae tumors: A frontier in endoscopic endonasal surgery-description of the surgical anatomy of the approach and report of illustrative cases. Int J Environ Res Public Health. (2022) 19:6413. doi: 10.3390/ijerph19116413 35681999 PMC9180479

[31] Moher D Liberati A Tetzlaff J Altman DG PRISMA Group. Preferred reporting items for systematic reviews and meta-analyses: the PRISMA statement. Ann Intern Med. (2009) 151:264–269, W64. doi: 10.7326/0003-4819-151-4-200908180-00135 19622511

[32] Al-Sheibani S Zanation AM Carrau RL Prevedello DM Prokopakis EP McLaughlin N. Endoscopic endonasal transpterygoid nasopharyngectomy. Laryngoscope. (2011) 121:2081–9. doi: 10.1002/lary.22165 21898447

[33] Battaglia P Turri-Zanoni M Lepera D Sica E Karligkiotis A Dallan I. Endoscopic transnasal approaches to pterygopalatine fossa tumors. Head Neck. (2016) 38 Suppl 1:E214–220. doi: 10.1002/hed.23972 25536922

[34] Bignami M Pietrobon G Arosio AD Fazio E Nocchi Cardim L Strocchi S. Juvenile angiofibroma: what is on stage? Laryngoscope. (2022) 132:1160–5. doi: 10.1002/lary.29801 34374999

[35] Borghei P Baradaranfar MH Borghei SH Sokhandon F. Transnasal endoscopic resection of juvenile nasopharyngeal angiofibroma without preoperative embolization. Ear Nose Throat J. (2006) 85:740–743, 746. doi: 10.1177/014556130608501114 17168151

[36] El Morsy SM Khafagy YW. Transnasal endoscopic management of angiofibroma extending to pterygopalatine and infratemporal fossae. J Laryngol Otol. (2011) 125:701–5. doi: 10.1017/S0022215111000673 21693074

[37] Epprecht L Mosimann M Vital D Holzmann D. Morbidity and volumetric progression in juvenile nasopharyngeal angiofibroma in a long-term follow-up. J Neurol Surg B Skull Base. (2018) 79:533–7. doi: 10.1055/s-0038-1635255 PMC6239873 30456021

[38] Fyrmpas G Konstantinidis I Constantinidis J. Endoscopic treatment of juvenile nasopharyngeal angiofibromas: our experience and review of the literature. Eur Arch Otorhinolaryngol. (2012) 269:523–9. doi: 10.1007/s00405-011-1708-6 21789677

[39] Gupta AK Rajiniganth MG Gupta AK. Endoscopic approach to juvenile nasopharyngeal angiofibroma: our experience at a tertiary care centre. J Laryngol Otol. (2008) 122:1185–9. doi: 10.1017/S002221510700148X 18394208

[40] Gupta DP Gupta S Shreevidya SR. Endoscopic modified Denker’s approach for the treatment of juvenile nasopharyngeal angiofibroma. Indian J Otolaryngol Head Neck Surg. (2022) 74:921–8. doi: 10.1007/s12070-020-01984-w PMC9702475 36452834

[41] Hofmann T Bernal-Sprekelsen M Koele W Reittner P Klein E Stammberger H. Endoscopic resection of juvenile angiofibromas–long term results. Rhinology. (2005) 43:282–9. 16405273

[42] Hofstetter CP Singh A Anand VK Kacker A Schwartz TH. The endoscopic, endonasal, transmaxillary transpterygoid approach to the pterygopalatine fossa, infratemporal fossa, petrous apex, and the Meckel cave. J Neurosurg. (2010) 113:967–74. doi: 10.3171/2009.10.JNS09157 19929194

[43] Janakiram TN Sharma SB Panicker VB. Endoscopic excision of non-embolized juvenile nasopharyngeal angiofibroma: our technique. Indian J Otolaryngol Head Neck Surg. (2016) 68:263–9. doi: 10.1007/s12070-016-1013-1 PMC4961649 27508124

[44] Nicolai P Villaret AB Farina D Nadeau S Yakirevitch A Berlucchi M. Endoscopic surgery for juvenile angiofibroma: a critical review of indications after 46 cases. Am J Rhinol Allergy. (2010) 24:e67–72. doi: 10.2500/ajra.2010.24.3443 20338105

[45] Plzák J Kratochvil V Kešner A Šurda P Vlasák A Zvěřina E. Endoscopic endonasal approach for mass resection of the pterygopalatine fossa. Clinics (Sao Paulo). (2017) 72:554–61. doi: 10.6061/clinics/2017(09)06 PMC5629706 29069259

[46] Roger G Tran Ba Huy P Froehlich P Van Den Abbeele T Klossek J-M Serrano E. Exclusively endoscopic removal of juvenile nasopharyngeal angiofibroma: trends and limits. Arch Otolaryngol Head Neck Surg. (2002) 128:928–35. doi: 10.1001/archotol.128.8.928 12162773

[47] Shin M Shojima M Kondo K Hasegawa H Hanakita S Ito A. Endoscopic endonasal craniofacial surgery for recurrent skull base meningiomas involving the pterygopalatine fossa, the infratemporal fossa, the orbit, and the paranasal sinus. World Neurosurg. (2018) 112:e302–12. doi: 10.1016/j.wneu.2018.01.041 29339322

[48] Wormald PJ Van Hasselt A. Endoscopic removal of juvenile angiofibromas. Otolaryngol Head Neck Surg. (2003) 129:684–91. doi: 10.1016/s0194-5998(03)01580-8 14663436

[49] Wu X Pan LS Wu BW Wu J Chen YX Xie SH. Endoscopic endonasal approach for trigeminal schwannomas: tailored approaches based on lesion traits. Laryngoscope. (2023) 133:2564–71. doi: 10.1002/lary.30834 37341509

[50] Zhang Q Feng K Ge C Hongchuan G Mingchu L. Endoscopic endonasal management of trigeminal schwannomas extending into the infratemporal fossa. J Clin Neurosci. (2012) 19:862–5. doi: 10.1016/j.jocn.2011.09.023 22386480

[51] Zhou B Huang Q Shen P-H Cui S-J Wang C-S Li Y-C. The intranasal endoscopic removal of schwannoma of the pterygopalatine and infratemporal fossae via the prelacrimal recess approach. J Neurosurg. (2016) 124:1068–73. doi: 10.3171/2015.3.JNS132702 26339855

[52] Meher R Kathuria S Wadhwa V Ali MR Shah B Bansal A. Preoperative emobilisation of juvenile nasopharyngeal angiofibroma. Am J Otolaryngol. (2022) 43:103532. doi: 10.1016/j.amjoto.2022.103532 35714497

[53] Vinciguerra A Saccardo T Verillaud B Herman P. Extended endoscopic pre-lacrimal medial maxillectomy to the anterior maxillary sinus wall. Laryngoscope. (2023) 133:2874–7. doi: 10.1002/lary.30620 36861770

[54] El-Sayed I Pletcher S Russell M McDermott M Parsa A. Endoscopic anterior maxillotomy: infratemporal fossa via transnasal approach. Laryngoscope. (2011) 121:694–8. doi: 10.1002/lary.21469 21433014

[55] Nicolai P Berlucchi M Tomenzoli D Cappiello J Trimarchi M Maroldi R. Endoscopic surgery for juvenile angiofibroma: when and how. Laryngoscope. (2003) 113:775–82. doi: 10.1097/00005537-200305000-00003 12792310

[56] Yang L Hu L Zhao W Zhang H Liu Q Wang D. Endoscopic endonasal approach for trigeminal schwannomas: our experience of 39 patients in 10 years. Eur Arch Otorhinolaryngol. (2018) 275:735–41. doi: 10.1007/s00405-018-4871-1 29350272

[57] Zoli M Milanese L Bonfatti R Faustini-Fustini M Marucci G Tallini G. Clival chordomas: considerations after 16 years of endoscopic endonasal surgery. J Neurosurg. (2018) 128:329–38. doi: 10.3171/2016.11.JNS162082 28409727

[58] Pasquini E Sciarretta V Farneti G Ippolito A Mazzatenta D Frank G. Endoscopic endonasal approach for the treatment of benign schwannoma of the sinonasal tract and pterygopalatine fossa. Am J Rhinol. (2002) 16:113–8. doi: 10.1177/194589240201600208 12030357

[59] Zoli M Ratti S Guaraldi F Milanese L Pasquini E Frank G. Endoscopic endonasal approach to primitive Meckel’s cave tumors: a clinical series. Acta Neurochir (Wien). (2018) 160:2349–61. doi: 10.1007/s00701-018-3708-4 30382359

[60] Diaz A Wang E Bujnowski D Arimoto R Armstrong M Cyberski T. Embolization in juvenile nasopharyngeal angiofibroma surgery: A systematic review and meta-analysis. Laryngoscope. (2023) 133:1529–39. doi: 10.1002/lary.30616 36789781

[61] Falcon RT Rivera-Serrano CM Miranda JF Prevedello DM Snyderman CH Kassam AB. Endoscopic endonasal dissection of the infratemporal fossa: Anatomic relationships and importance of eustachian tube in the endoscopic skull base surgery. Laryngoscope. (2011) 121:31–41. doi: 10.1002/lary.21341 21181982

[62] Watanabe K Passeri T Hanakita S Giammattei L Zomorodi AR Fava A. Extradural anterior temporal fossa approach to the paranasal sinuses, nasal cavities through the anterolateral and anteromedial triangles: Combined microscopic and endoscopic strategy. Acta Neurochir (Wien). (2021) 163:2165–75. doi: 10.1007/s00701-021-04850-y 33914166

