# Supplementary material for: Lessons learned in 20 years of endoscopic endonasal surgery for pterygopalatine and infratemporal fossae lesions: analysis of a patient series and systematic review of literature
Source: Front Oncol. 2025 Jun 17;15:1568913. doi: 10.3389/fonc.2025.1568913 (PMC12209365; doi:10.3389/fonc.2025.1568913)
Supplement: Supplementary file 1 [file Table1.docx]

**Supplementary Tables**

**Suppl. Table 1**. Histotypes included in the “other malignant tumors” category

| Other malignancies |  |  |
| --- | --- | --- |
| Embrionic C. | 1 | 1.0% |
| Epimyoepithelial C. | 1 | 1.0% |
| Lymphoepithelial C. EBV+ | 2 | 2.0% |
| Myoepithelial C. | 1 | 1.0% |
| Neuroendocrine C. | 1 | 1.0% |
| Non-cheratinic indifferenziate C. | 1 | 1.0% |
| Sarcomatoid C | 1 | 1.0% |
| Squamous C. | 6 | 6.0% |
| ITAC | 2 | 2.0% |
| Secondarism | 1 | 1.0% |
| Ewing S. | 1 | 1.0% |

Legend

C.: carcinoma; EBV: Epstein-Barr Virus; ITAC: intestinal type adenocarcinoma; S.: sarcoma.
